# An Interactive Communication Model for Self-Care—Regardless of Health Literacy: Protocol for a Quasi-Experimental Study

**DOI:** 10.2196/37673

**Published:** 2023-03-31

**Authors:** Lisa Korsbakke Emtekaer Haesum, Ole Kristian Hejlesen, Flemming Witt Udsen

**Affiliations:** 1 Department of Nursing, University College of Northern Denmark Aalborg Denmark; 2 Department of Health Science and Technology, Faculty of Medicine, Aalborg University Aalborg Denmark

**Keywords:** health literacy, interactive communication, nursing, quasi-experimental study, economic evaluation, economic, cost, rehabilitation, primary care, nurse, communication, health information, patient education, health resource

## Abstract

**Background:**

Clear dialogue-based (interactive) communication that ensures comprehension and recall becomes more important in patient-provider interactions, especially in relation to patients with chronic diseases, where self-management education and counseling are cornerstones in managing these diseases. If patients with chronic disease experience challenges in obtaining, understanding, and applying health-related information (necessary to make informed health decisions and sufficiently manage their health), clear communication and ensuring comprehension become even more critical in the patient-provider interactions. Furthermore, patient-provider communication has been proposed as a potential pathway through which health literacy might influence health outcomes, especially in individuals with chronic diseases. Hence, adjusting communication to the individual level of health literacy might have a positive influence on health outcomes. On this basis, the authors have developed a web-based interactive communication model that both seeks to accommodate health literacy by allowing tailored communication and ensure comprehension and recall between nurses and patients.

**Objective:**

This study seeks to examine the use of an IT solution that comprises an interactive communication model that seeks to accommodate health literacy in communication and ensure comprehension and recall between nurses and patients.

**Methods:**

A quasi-experimental control group study including full economic evaluation with 6-month follow-up. Based on power calculation, a total of 82 participants will be included. Participants are assigned either the interactive communication model (intervention) or usual nursing care. It will be assessed if the model influences the level of health literacy and participants experience a higher health-related quality of life. Further, cost-effectiveness will be evaluated. Overall, the statistical methods will follow an intention-to-treat principle. Results will be presented in accordance with the Transparent Reporting of Evaluations with Non-randomized Designs guidelines for nonrandomized designs as well as the Consolidated Health Economic Evaluation Reporting Standards.

**Results:**

This paper describes a protocol for a clustered quasi-experimental control study that seeks to evaluate the effectiveness of the interactive communicative model. Most studies in the field of health literacy are epidemiological studies that seek to address the effects of poor health literacy in populations and its potential impact on health inequity. A total of 82 participants, who receive community nursing will be included. The final trial day is May 1, 2022, with the first report of results in the final quarter of 2022.

**Conclusions:**

The results of the trial can create the base for conducting a large-scale study and inspire the conduction of more studies that seeks to create and evaluate interventions aimed at enhancing the level of health literacy and reducing the usage of health resources.

**Trial Registration:**

ClinicalTrials.gov NCT04929314; https://clinicaltrials.gov/ct2/show/NCT04929314

**International Registered Report Identifier (IRRID):**

DERR1-10.2196/37673

## Introduction

### Background

The demographic profile is changing all over the world with a longer life expectancy that yields an increase in the number of people living with chronic diseases [1]. The challenge of meeting the needs of this growing number of people will fall upon already overwhelmed health care services that are struggling to cope with the current demands of changing health care systems [2]. As a result, the role of modern patients is changing from paternalistic models of health care that placed the patient as a passive recipient to more active involvement of patients in their own care [3]. This change in the role of modern patients, however, sets some requirements to their level of patient empowerment and self-management; two concepts wherein health literacy (HL) plays an important role [4]. HL is a relatively new area of research that focuses on the ability to make sensible health choices that promote and maintain good health, based on the individual ability to access, evaluate, and use health-related information [5]. The World Health Organization has defined HL as “the cognitive and social skills which determine the motivation and ability of individuals to gain access to, understand and use information in ways which promote and maintain good health” [6].

The concept of HL includes multiple levels with different requirements to personal skills in relation to health care, making it a very broad and complex concept. In a widely used approach, Nutbeam [7] frames the different levels of HL as follows:

- *A functional level of HL* comprises the basic skills of numeracy, reading and writing that allow an individual to function effectively in a health care setting (eg, by being able to navigate the health care system, take the right dose of medication, and provide an accurate presentation of symptoms).
- *An interactive level of HL* refers to a more advanced level of literacy, that is, social and cognitive skills that allow an individual the capability to understand and retrieve health information. At this level, individuals are also able to engage actively in a dialog about their disease and course of treatment with health care professionals.
- *The critical level of HL* comprises an advanced set of skills that allow the capability to critically analyze health-related information retrieved from the health care system or independently and the ability to act on this information.

According to a Danish study [8], 10%-20% of the Danish population experience difficulties with the ability to understand health-related information sufficiently to act on it and the ability to interact with health care professionals (2 important dimensions of HL). Hence, they do not have the necessary HL competencies to manage their health sufficiently, which incur a higher risk of adverse health effects, including improper use of health care services and medications, poor self-management, and poor health outcomes [9]. On this basis, clear communication and ensuring comprehension and recall become more important in patient-provider interactions, especially in relation to patients with chronic diseases, where self-management education and counseling are cornerstones in managing these diseases. If patients with chronic disease also experience challenges in obtaining, understanding, and applying health-related information (necessary to make informed health decisions and sufficiently manage their health), clear communication and ensuring comprehension become even more critical in the patient-provider interactions [10]. Furthermore, patient-provider communication has been proposed as a potential pathway through which HL might influence health outcomes, especially in individuals with chronic diseases. Hence, adjusting communication to the individual level of HL might have a positive influence on health outcomes [11,12]. Nurses play an essential role in providing education and counseling for patients with a chronic disease through communication—it is considered a core competency in their nursing practice [13]. However, nurses rarely use communication that focuses on comprehension and recall in their interaction with patients [14]. On this basis, this study examines the use of an interactive communication model (ICM) that seeks to accommodate HL in communication and ensure comprehension and recall between nurses and patients.

### Objectives

The main objective of this study is to conduct a broad evaluation of a web-based ICM (refers to the interaction between the user [the community nurses in this study] and the recipients [the citizens receiving community nursing in this study]) that both seek to identify knowledge and skills (HL) by enhancing tailored communication and ensure recall and comprehension between patients and nurses. Specific research questions concern whether the ICM improves HL among participants, who receive community nursing care, and whether they experience a higher health-related quality of life. Secondary outcomes include evaluating the cost-effectiveness of the ICM and assessing if it improves the quality of community nursing care, which includes the evaluation of citizens’ adherence to care and treatment as well as the community nurses’ satisfaction with using the ICM in their care. Finally, how participants, who receive community nursing care from the nurses in the intervention group, experience nursing care prior and after the intervention was assessed.

## Methods

### Study Design

This trial is conducted as a clustered quasi-experimental control group study with 6-month follow-up. The trial runs from November 2021 to May 2022. This study was originally designed as a 2-armed cluster-randomized controlled trial. However, the randomization protocol was abandoned after several months of unsuccessful recruitment efforts, and the study was redesigned as a quasi-experimental control group study. The primary reason for abandoning the randomized controlled trial design was challenges related to recruiting all districts making it difficult to conduct a meaningful randomization. The trial is registered at ClinicalTrials.gov (NCT04929314).

### Ethical Considerations

The trial has been presented to the Regional Ethical Committee for Medical Research in North Denmark. The committee determined that no ethical approval was necessary (number 2020-000992-45). The trial has been approved by the Danish Data Protection Agency. Data will be anonymized and stored in accordance with the Danish Data Protection Rules [15].

The investigation conforms with the principles outlined in the Declaration of Helsinki. Potential participants were informed of the content and aims of the study and required to give written, informed consent before enrollment. All participants were informed that the data collected can be used for research purposes, and they were asked to sign an informed written consent form if they wished to participate after thoroughly reviewing the material regarding the study. The informed written consent includes consent for publication. All participants were informed that participation is voluntary, and they can withdraw from the study at any time. The participants did not receive any compensation for their participation in the study.

### The Target Sample

The trial population consists of citizens in 4 districts in the municipality of Aalborg who receive health care services. Participants are eligible for inclusion if they receive home health care (community nursing at least every 2 weeks) in Aalborg Municipality and are older than 18 years at the point of enrollment. Further, participants must receive community health care services for a period of more than 3 months prior to enrollment and be able to speak, read, and write Danish. The participants must also have a fixed residence in Aalborg Municipality. Participants will be excluded from the study if they do not meet the inclusion criteria and if they are terminal or diagnosed with a psychiatric disease, dementia, or other severe cognitive impairments. Participants are also excluded if they are unable to understand and speak Danish (unable to complete study questionnaires).

### Recruitment

**Figure 1:**
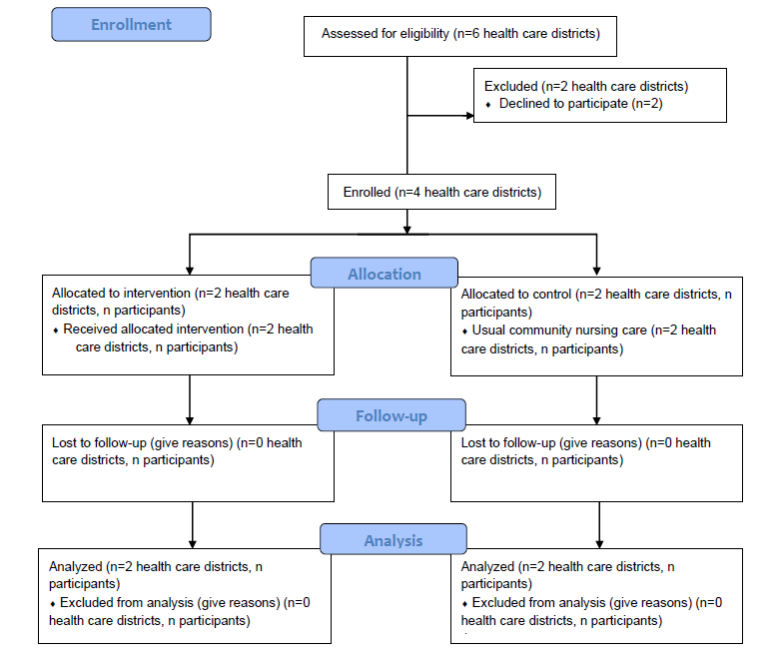
Flowchart diagram representing the design of the study.

Recruitment has been ongoing since April 2021 in 4 out of 6 care districts in Aalborg Municipality. The community nurses identify eligible participants (Figure 1) and create a list of potential participants, who have given permission for the researcher to contact them by telephone. The researcher provides further information about the trial, answers questions, and guides them regarding the questionnaires and the written consent form. Subsequently, written information about the study, questionnaires, and a written consent form are sent to eligible participants after receiving oral consent over the telephone. If the participants still wish to participate after thoroughly reviewing the material received, they will return the written consent form and the questionnaires to the community nurse before the date specified in the material. The researcher’s contact information is highlighted in the written information, allowing the participants an opportunity to receive support in filling out the questionnaires. Due to the COVID-19 situation, it is attempted to reduce physical contact between the researcher and the participants, and that is why all communication is preferred over the telephone.

### The Intervention

#### Overview

The intervention is based on the research-based knowledge about HL and focuses on interactive communication between community nurses and citizens who receive community nursing care. It seeks to ensure uniform interactive communication from the community nurses to ensure that citizens understand and adapt the information given by nurses, regardless of the level of HL. The intervention is not based on research alone but has been developed in close collaboration with practice (Aalborg Municipality). Almost all community nurses in Aalborg Municipality have participated in a workshop on rehabilitation, self-rehabilitation, and HL. In total, 14 workshops were conducted (a total of 335 community nurses from Aalborg Municipality participated) in the autumn of 2019 as part of a pilot project prior to this research project. A central point of these workshops was to identify challenges related to the more active involvement of citizens (receiving community nursing care) in their own health and disease. Aalborg Municipality puts great emphasis on “self-rehabilitation,” where the ambition is to transfer as many health-related tasks (eg, medication administration and dispensing) as possible to the citizens receiving community nursing care and, thus, placing more responsibility on the citizens themselves. The development of the intervention is based on a best-practice approach from the literature combined with solid experience from nursing practice. The involvement of the community nurses in the development of the intervention has also contributed to a sense of ownership among the users (the community nurses in Aalborg Municipality). It should be noted that the community nurses have never seen or used the intervention during the workshops; the workshops contributed to the development of the intervention, as it was identified what community nurses saw as challenges in strengthening self-care among their citizens.

**Figure 2:**
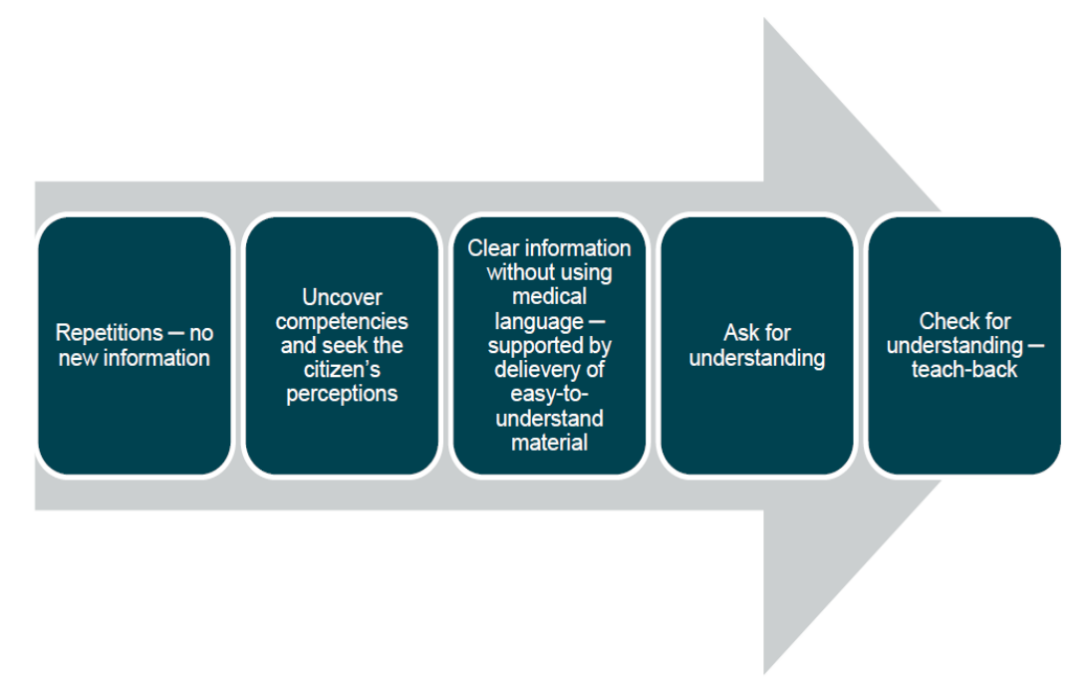
Illustration of the elements in the interactive communication model (ICM). The model is inspired by Schillinger et al [17].

The material from the workshops, in conjunction with relevant research literature, has formed the basis for the development of an intervention containing the following components: (1) a development of competences is related to HL among community nurses in Aalborg Municipality (only those who are responsible for citizens who receive the intervention). The community nurses will receive general education about HL and how to screen for it with the Danish European Health Literacy Survey Questionnaire (HLS-EU-Q-16) [16]. It should be noted that assessing each citizen’s level of HL is only being done as part of the study and not as part of standard protocol. (2) The community nurses will also receive practical training in systematic and integrative communication using the ICM with integrated teach-back, see Figure 2 for detailed description. The systematic and integrative communication is supplemented by the delivery of easy-to-understand material to the citizen. The material has been developed and evaluated in advance. (3) The community nurses will be introduced to a guide that describes how to document the use of the ICM. This guide seeks to ensure consistency and uniformity in the documentation of nursing care.

#### Repetitions

The model starts with a repetition of key points (very few sentences) from the last visit or learning session. The community nurse simply repeats key points and provides no new information at this stage. By the first visit, the community nurse cannot perform repetitions, but instead, the citizen is presented with the plan to enhance knowledge and self-care.

#### Uncover Competencies and Seek the Citizen’s Perceptions

This allows the community nurse and citizen to reach a common understanding. Moreover, it helps the community nurse assess how to tailor information and instructions for the citizen.

#### Clear Information Without Using Medical Language

The community nurse provides information and instructions at a very easy-to-understand level without using medical language. The information and instructions are provided based on predefined material made available to the community nurse on a web page accessible from her working tablet. The information and instructions are supplemented by the delivery of easy-to-understand material to the citizen.

#### Ask for Understanding

The community nurse asks the citizen directly if they have understood the given information and what has been discussed.

#### Check for Understanding—Teach-Back

The community nurse uses the teach-back technique to ensure that the citizen understands the information or instructions and what has been discussed. The community nurse asks the citizen to retell (in their own words) the information and instructions provided. This stage may also involve the citizen having to demonstrate the performance of a “task,” for example, the community nurse asks the citizen, with chronic obstructive pulmonary disease, to demonstrate their inhalation technique.

The ICM must be an easily accessible and efficient tool for community nurses, and it is therefore created as a web-based IT solution. The IT solution is entitled “personalized support for self-care” and can be accessed from the community nurses’ working tablet—direct link on the start page.

The content of the IT solution is selected based on the most prominent diseases and reasons for receiving community nursing in Northern Jutland. People receiving community nursing often have multiple health-related challenges and comorbidities and have a high level of vulnerability, which sets high requirements to their level of HL. The concept is that the ICM (made available in the web-based IT solution) can support community nurses in accommodating citizens’ broad range of information and communication preferences. The ICM uncovers what people know about their own health and situation instead of a subjective assumption of what they know; thus, it can be considered a more objective approach to accessing knowledge and skills. The IT solution includes a general description of the ICM and how to conduct each individual communicative step in the model. The general description is supported by a graphical illustration of the different steps in the model. There are thorough instructions on how to incorporate the different communicative steps into practice; a “correct” use can promote more interactive communication.

The IT solution also describes how to use the ICM in relation to the most prominent diseases such as chronic obstructive pulmonary disease, diabetes, and cardiovascular diseases. It is thoroughly described how to carry out each individual step in the ICM related to the disease in question—all the way down to how questions should be formulated and asked to uncover citizens’ knowledge and level of skills (step 2—ask for understanding) and how information is provided in a concise easy-to-understand manner without medical language (step 3—clear information without using medical language). The idea is that users of the IT solution, containing the ICM, do not have to decide for themselves what easy-to-understand language is because it is provided by the web-based system. The instructions and information formulated in the IT solution, containing the ICM, have been developed in the following manner: information and content about the specific diseases are inspired by an acknowledged national patient–centered web page (sundhed.dk) and use of pedagogical tools that focus on increasing and ensuring the understanding with the recipient [18,19]. The material in the IT solution, containing the ICM, is kept on a readability of sixth- to seventh-grade level and, thus, users provide information and guidance on this level when using the ICM. The material was read thoroughly by independent reviewers to ensure easy-to-understand and concise language not exceeding sixth- to seventh-grade level. It should be noted that the IT solution also provides different links and video material (easy-to-understand that can be used in *step 3—clear information without medical language*) relevant to the specific diseases.

Even though the instructions and information in the IT solution, containing the ICM, serve as a guide to learn the ICM technique, the goal is that users adapt the communication technique into their everyday practice. The IT solution, containing the ICM, is intended to facilitate the use of a very easy-to-understand language and check the understanding of information by the recipient. The community nurses in this study, who are responsible for citizens who receive the intervention, will be instructed to use the IT solution, containing the ICM, at each visit. The web-based IT solution, containing the ICM, has been tested in a small pilot study [20].

### The Control: Usual Community Nursing Care

Danish community nursing care combines nursing practice and primary health care in the Danish community setting. Denmark comprises 98 municipalities, which have a specialized role in community nursing care that requires nursing and practical help in the citizens’ own homes. Danish community nursing care varies across the municipalities. Community nurses primarily deliver health services, health education, preventive care, rehabilitation, etc, to Danish citizens. Community care nurses in the control group will not receive the special training described earlier, and the participants in this group will receive their usual care and treatment throughout the study period of 6 months.

### Allocation

Aalborg Municipality is comprised of 6 health care districts. However, due to practical reasons, a total of 2 districts declined to participate in this study. The remaining 4 districts are matched 1:1 in terms of geographical area (2 rural districts are matched and so are 2 urban), and each pair is then allocated so that one district is in the intervention and the other is in the control group. Coincidently, each pair also matches with the estimated level of social classes (judged by the municipality). Before allocation, all participants must have signed informed consent and conducted baseline measurements.

### Outcome Measurements

#### Primary and Secondary Outcomes

The primary outcome of this study is the change in the level of HL assessed by the HLS-EU-Q-16 questionnaire [16] from baseline to follow-up at 6 months. The HLS-EU-Q-16 measures self-perceived HL and reflects the conformity between individual competencies needed to navigate the health care system and situational complexities. Hence, the HLS-EU-Q-16 reflects the self-perceived level of HL, and this should be taken into consideration when interpreting the survey results. This study hypothesizes that adding the ICM and continuity in documentation to usual community care will lead to a significantly higher level of self-perceived HL for the included participants.

Two secondary outcomes will be reported: first, the between-group difference in health-related quality of life measured by the EQ-5D-3L questionnaire [21] at follow-up after 6 months, and second, the incremental cost-effectiveness ratio representing the total cost per additional HLS-EU-Q-16 point accrued from baseline to follow-up at 6 months. This trial adopts a health- and social sector perspective with the following cost categories: intervention costs, costs associated with all hospital contacts, primary sector costs (ie, general practitioners and other primary care visits), costs associated with home nurse visits, and home care costs due to individual care and practical help as well as individual or group rehabilitation costs. Service cost components will be based on register data, that is, from the Danish National Patient Register (all hospital contacts) [22], the National Health Insurance Service Register (all primary care contacts) [23], and the electronic care register in Aalborg Municipality. All data can be unambiguously linked with the help of a 10-digit social security number that all Danish citizens have.

#### Additional Outcomes

The quality of nursing care is assessed using a self-constructed questionnaire, which has been used in a previous pilot study by our research group. The quality of nursing care will be evaluated by the community nurses in Aalborg Municipality.

Another additional outcome is to assess how the citizens, who receive community nursing care from the nurses in the intervention group, experience nursing care prior to the intervention and after the intervention. Hence, the citizens will be interviewed twice during the study: prior to the intervention and after the intervention. As per the study by Kvale and Brinkmann [24], semistructured qualitative individual face-to-face interviews will be conducted with the citizens in the intervention group in the comfort of the own homes. The interviews will be conducted before the community nurses in the intervention group receive any special training and start using the intervention (prior) and again at follow-up (after). The interviews will be recorded and transcribed by researchers.

It is hypothesized that the ICM will be associated with a larger gain in HL in the intervention group, as the model is expected to increase citizens’ control of their own lives as it establishes a more solid knowledge and empowerment of their disease and health [25-27].

### Sample Size and Power Calculation

Sample size calculation for the primary outcome was performed in Stata (version 16; StataCorp). The intention is to detect a standardized mean difference in favor of the intervention based on Cohen *d*>0.8 [28]. Requiring a power of 80% and a significance level (1-sided) of 0.05, with 4 clusters given (2 in each alternative) and assuming a coefficient of variation in cluster size of 0.25 and intracluster correlation equal to 0.05, the required sample size is 37 participants in each arm (or an average of 18.5 participants in each cluster). With an expected loss-to-follow-up of 10%, the total required sample size is 41 in each arm (or an average of 20.5 in each cluster).

### Analyses

Overall, the statistical methods will follow an intention-to-treat principle [29]. Results from the trial will be presented in accordance with the Transparent Reporting of Evaluations with Non-randomized Designs guidelines for nonrandomized designs [30] as well as the Consolidated Health Economic Evaluation Reporting Standards [31]. Missing data will be subject to multiple imputations [32]. Data will be analyzed with Stata 16 software.

Between-group differences for both the primary and secondary outcomes of health-related quality of life and cost-effectiveness will be calculated by applying multilevel generalized linear models, and both an unadjusted and adjusted estimate controlling for meaningful baseline differences will be reported. To allow for evaluation of the impact of variability and parameter uncertainty, the economic evaluation will also report the results of one-way and probabilistic sensitivity analysis [33]. The probabilistic sensitivity analysis will be carried out using Excel (Microsoft Inc).

All data from the interviews will be analyzed using NVivo R1 (version 13.0; Lumivero). Inspired by Kvale and Brinkmann [24], the analysis will be conducted according to the following steps: (1) identification of key themes and definitions from theoretical framework, inspired by Braun and Clarke [34]. (2) Review of all data from the interviews to obtain an overall impression of the themes on how the citizens experience their level of HL and self-management, both prior to the intervention and after the intervention. (3) Design of code tree based on key definitions and concepts derived from the theoretical framework and interviews. (4) The researchers will discuss and revise the code tree to ensure intersubjectivity. (5) The first step of the coding process will be centered on gaining an overall understanding of the citizens’ perception of their level of HL and self-management, both prior to the intervention and after the intervention. (6) The next step will focus on widening the understanding beyond the citizens’ perception. What presents a challenge to their level of HL and self-management and what strengthens it? (7) The final step will be an analysis centered on the citizens’ perception of their level of HL and self-management and if this is altered when community nurses use the intervention in their nursing care. (8) Data will be condensed and presented as key themes and findings.

## Results

A total of 82 participants, who receive community nursing will be included. The final trial day is May 1, 2022, with the first report of results in the final quarter of 2022.

## Discussion

This paper describes a protocol for a clustered quasi-experimental control study that seeks to evaluate the effectiveness of an IT solution that comprises the ICM seeking to enhance more dialogue-based and tailored communication. Most studies in the field of HL are epidemiological studies that seek to address the effects of poor HL in populations and its potential impact on health inequity [35]. There is, however, a call for studies centered on developing interventions aimed at strengthening HL; strengthening HL in populations and making health services more accessible to people with a low level of HL may be a practical strategy to reduce disparities and promote greater equity in health [9,36]. To our knowledge, this is one of the first studies to assess the effectiveness of an intervention that seeks to enhance the level of HL. The strength of this study is that it attempts to illuminate the effects of an intervention centered on interactive communication using a control group design. Another strength is that the study has characteristics like an effectiveness trial, as it evaluates the effects of enhancing knowledge of HL among community care nurses and integrating the use of the ICM in their nursing care.

A limitation of this study is the design as a quasi-experimental and abandoning the original randomization protocol. This means that this study may be subjected to selection bias, as between-group baseline characteristics may differentiate. Another limitation is the relatively short follow-up time, as between-group comparisons can only be made at the 6-month follow-up. This might be problematic since changes in particularly costs and health-related quality of life require behavior change that might take time to occur. However, recent guidance for developing and evaluating complex interventions recommends that broad-ranging evaluations are conducted in the early phases and that they also include economic evaluation. The aim is to identify the scope of cost and different effects that can help decision makers understand the potential of the intervention and decide whether proceeding to a large-scale evaluation is worthwhile [37]. In making this judgment, it is a standard health economic practice also to model the joint decision uncertainty surrounding particularly health-related quality of life outcomes and costs [38,39]. Reporting cost-effectiveness (including health-related quality of life, costs, or quality-adjusted life years) with 6-month follow-up specific to the domain of HL has also previously been reported [40,41] and to other interventions aimed at changing behavior [42,43]. The expectation is that the results of the trial therefore can create the base for deciding to conduct a large-scale study and inspire the conduction of more studies that seeks to create and evaluate interventions aimed at enhancing the level of HL and reducing the usage of health resources.

## Abbreviations

HL: health literacy
HLS-EU-Q-16: European Health Literacy Survey Questionnaire
ICM: interactive communication model

## Data Availability

All data will be stored in a secure drive (UCNemDok). Only researchers from the University College of Northern Denmark and Aalborg University will have access to the trial data set.
